# Is polyphagy of a specific cryptic *Bemisia tabaci* species driving the high whitefly populations on cassava in eastern Africa?

**DOI:** 10.1007/s10340-024-01832-8

**Published:** 2024-09-02

**Authors:** Annet Namuddu, Osnat Malka, Susan Seal, Sharon van Brunschot, Richard Kabaalu, Christopher Omongo, Shai Morin, John Colvin

**Affiliations:** 1 Natural Resources Institute, Central Avenue, Chatham Maritime, Kent, ME4 4TB UK; 2 National Crops Resources Research Institute, P.O. Box 7084, Kampala, Uganda; 3 Department of Entomology, The Hebrew University of Jerusalem, P. O. Box 12, Rehovot, Israel; 4 CSIRO Health and Biosecurity, Brisbane, QLD Australia; 5 The Gaza Envelope Research and Development Center, Netivot, Israel

**Keywords:** Cryptic species, *MtCO1*, Host range, SSA1-SG1, MED-ASL

## Abstract

Since the 1990s, the cryptic whitefly (*Bemisia tabaci*) has been linked to severe viral disease pandemics affecting cassava, a crucial staple crop in eastern Africa. This surge in whitefly populations has also been observed in other crops and uncultivated plants. While previous surveys have connected the increase on cassava to two specific populations, SSA1 and SSA2, the dynamics behind the population growth on other plants remain unclear. Additionally, other *B*. *tabaci* species, including EA1, IO, MED, SSA9, and SSA10, have been found on cassava in smaller numbers. This study aimed to identify the host plants that support the growth and development of different *B*. *tabaci* in Uganda by collecting fourth-instar nymphs from cassava and 20 other common host plants. Host transfer experiments were conducted to test the ability of seven species (EA1, MEAM1, MED-Africa Silver Leafing (ASL), SSA1-subgroup1, SSA1-Hoslundia, SSA6, and SSA12) to develop on cassava. The identities of the nymphs were determined using partial *mitochondrial cytochrome oxidase 1* sequences. Twelve *B*. *tabaci* species were identified, including two novel species, based on the 3.5% nucleotide sequence divergence. Cassava was colonised by SSA1-SG1, SSA1-SG2, and SSA2. The most prevalent species were SSA1-SG1, MED-ASL, and SSA13, which were also the most polyphagous, colonising multiple plant species. Several whitefly species colonised specific weeds, such as *Aspilia africana* and *Commelina benghalensis*. The polyphagous nature of these species supports continuous habitats and virus reservoirs. Effective management of whitefly populations in eastern Africa requires an integrated approach that considers their polyphagy and the environmental factors sustaining host plants.

**Supplementary Information:**

The online version contains supplementary material available at 10.1007/s10340-024-01832-8.

## Introduction

*Bemisia tabaci* (Gennadius) is a group of cryptic species, whose members differ in their levels of polyphagy (Dinsdale et al. 2010; Mugerwa et al. 2018; Vyskočilová et al. 2019). *B. tabaci* Middle East-Asia Minor1 (MEAM1) and Mediterranean (MED), for example, are highly polyphagous (Brown et al. 1995; Shah and Liu 2013; Xia et al. 2017), whereas some species have a limited host range. Examples of monophagous species include the Jatropha race, which colonises *Jatropha gossypifolia* L. in Puerto Rico (Bird 1957) and sub-Saharan Africa (SSA) 6 that colonises *Ocimum gratissimum* L. in Uganda (Sseruwagi et al. 2005). Polyphagous insect pests utilise various host plants from different plant families, which allows them to adapt to changes in agroecosystems such as senescence of preferred host plants, harvest, and changing cropping patterns (Kennedy and Storer 2000; Sivakoff et al. 2013). Therefore, knowledge of the host plant range and level of polyphagy of *B*. *tabaci* species is important for the development of effective management strategies of these pests. In addition, the choice of host plants determines the pathways of spread of whitefly-transmitted viruses (Polston and Capobianco 2013) and a wider vector host plant range may increase transmission of viruses to crops.

African cassava *B. tabaci*, namely SSA1 and SSA2, have been reported to be the most frequent species colonising cassava in Uganda (Legg et al. 2002, 2014; Sseruwagi et al. 2006; Mugerwa et al. 2018). Other *B. tabaci* species such as SSA9, SSA10, MED, Indian Ocean (IO) and East Africa (EA) 1 have also been collected from cassava, although in relatively low numbers (Legg et al. 2014; Mugerwa et al. 2018). Studies showed that the globally invasive whitefly species MEAM1 and MED (De Barro et al. 2011; Wang et al. 2011) occurred mainly on non-cassava host plants in Uganda, with MED being more prevalent than MEAM1 (Sseruwagi et al. 2005; Mugerwa et al. 2018; Namuddu et al. 2023). Adult whitefly surveys showed that the SSA1, particularly the subgroup1 (SSA1-SG1), was the most abundant cassava-feeding *B. tabaci* and it occurred on more than 30 plant species (Legg et al. 2014; Mugerwa et al. 2018; Mugerwa et al. 2021). However, adults are of limited reliability when studying the actual host plant range of *B. tabaci*, because their occurrence on a plant may not necessarily signify that it is good for nymphal growth and development (Sseruwagi et al. 2006). The adults, for example, could have been casually on the plant, searching for a suitable host for feeding and oviposition (van Lenteren and Noldus 1990; Walling 2008) at the time of collection.

When fourth-instar nymphs were collected from field host plants, Sseruwagi et al. (2006) revealed that the SSA1 could develop on five additional host plant species commonly found adjacent to cassava fields. These were: (i) *Manihot glaziovii* (Müll.Arg.) Allem, (ii) *J*. *gossypifolia*, (iii) *Euphorbia heterophylla* L., (iv) *Aspilia africana* (Pers.) C. D. Adams, and (v) *Abelmoschus esculentus* L.*.* In addition to collecting fourth-instar nymphs from alternative host plants in close proximity to cassava, it is also important to collect the fourth-instar nymphs from the non-cultivated host plants, in the surrounding vegetation near cassava. This would help to establish if they are the source of whiteflies that infest cassava when it is planted in a new field, facilitating the continuous high whitefly populations on cassava. It was hypothesised that highly fecund *B*. *tabaci* species could displace other whitefly species from cassava. In this study, host transfer experiments for some of the *B*. *tabaci* species that have been collected from cassava in low numbers were conducted to determine their ability to colonise cassava. The aim of this work, therefore, was to carry out a comprehensive investigation into the cassava-colonising *B. tabaci* species in Uganda and the alternative host plant species suitable for their feeding and development.

## Materials and methods

### Collection of fourth-instar nymphs within and adjacent to cassava fields

Fourth-instar *B. tabaci* nymphs were collected from cassava and eight cultivated plant species that are commonly intercropped or grown in close proximity to cassava and 13 non-cultivated plant species usually found within or adjacent to cassava fields (Table 1). Plants adjacent to cassava fields were defined as those positioned in a margin of 0–25 m away from the edges of the nearest cassava field. At least 10 nymphs were collected from two to three leaves of a given plant species and preserved in 90% ethanol in 1.5-mL Eppendorf tubes. The unparasitised fourth-instar nymphs were collected with the aid of a Peak 1966 Light Loupe 10 × hand lens from plants without virus symptoms.

**Table 1:** Host plant species from which whitefly colonies were collected, established and maintained

Common name	Botanical name	Propagation method	Collection site	Whitefly species
African basil	*Ocimum gratissimum*	Seed	Namulonge	SSA6
Beans	*Phaseolus vulgaris*	Seed	Namulonge	MEAM1
Bitter leaf	*Vernonia amygdalina*	Seed	Namulonge	SSA10*
Black-jack	*Bidens pilosa*	Seed	Namulonge	East Africa1
Cassava	*Manihot esculenta*	Cuttings	Namulonge	SSA2
Common wireweed	*Sida acuta*	Seed	Namulonge	SSA13*
Cotton	*Gossypium hirsutum*	Seed	Namulonge	MED-ASL
Cowpea	*Vigna unguiculata*	Seed	Kabanyolo	MED-ASL
Goatweed	*Ageratum conyzoides*	Seed	Namulonge	SSA1-Hoslundia*
Groundnuts	*Arachis hypogaea*	Seed	Namulonge	Unknown
Mexican fire plant	*Euphorbia heterophylla*	Seed	Namulonge	SSA13*
Muwugula	*Pavonia urens*	Seed	Namulonge	MED-ASL
Okra	*Abelmoschus esculentus*	Seed	Lira	MED-ASL
Orange bird-berry	*Hoslundia opposita*	Seed	Namulonge	SSA1-Hoslundia
Seed-under-leaf	*Phyllanthus niruri*	Seed	Namulonge	SSA1-SG1
Sweet potato	*Ipomoea batatas*	Cuttings	Namulonge	MED-ASL
Tickberry	*Lantana camara*	Seed	Namulonge	SSA13*
Tomato	*Solanum lycopersicum*	Seed	Namulonge	MEAM1
Tree cassava	*Manihot glaziovii*	Cuttings	Mukono	SSA1-SG2
Wandering Jew	*Commelina benghalensis*	Cuttings	Namulonge	SSA12
Wild sunflower	*Aspilia africana*	Seed	Namulonge	*Bemisia* Uganda1*

Underlined names are cultivated host plants, * whitefly that did not establish successful colonies at NRI. *SSA* sub-Saharan Africa; *SG* subgroup; *MED-ASL* Mediterranean African silver leafing; *MEAM1* Middle East-Asia Minor1

### Collection of fourth-instar nymphs from plants situated at least 300 m from cassava

Samples of fourth-instar *B. tabaci* nymphs on *A. africana*, *Phyllanthus niruri* L., *Hoslundia opposita* Vahl, *O*. *gratissimum*, *Vernonia amygdalina* Delile, *Lantana camara* L., *E*. *heterophylla* and *Sida acuta* Burm. f. in the vegetation situated > 300 m from cassava fields were collected for analysis. It proved difficult, however, to find a wide range of cultivated host plants with fourth-instar nymphs. Therefore, a field experiment was set up where the position of experimental plants could be manipulated. The non-cultivated plants species *Bidens pilosa* L., *Ageratum conyzoides* (L.) L., *Commelina benghalensis* L., *M. glaziovii*, *Pavonia urens* Cav. and cultivated ones, *V*. *unguiculata*, *A*. *hypogaea*, *P*. *vulgaris*, *Gossypium hirsutum* L., *Solanum lycopersicum* L., *A*. *esculentus* and *Ipomoea batatas* (L.) Lam. were set up in an experimental plot in an open field more than 300 m from cassava at the National Crops Resources Research Institute (NaCRRI), Namulonge, Uganda (N0.521257, E32.631322). Plants were grown in 3-L buckets in an insect-proof screen house until they had three to five fully expanded leaves. They were then transferred to the open field surrounded by a forest, shrubs and land that had been left to fallow. Prior to transferring them to the field, the buckets and plants were fully covered with plastic to avoid any infestation from cassava and/or sweet potato whiteflies during transportation. The area where the buckets were placed had the grass cut back every two weeks. Each plant species had six replicates, and each plant was assigned a random number. The plants were spaced at 1-m intervals in three rows in a 2 m × 2 m grid and allocated randomly to a position. They were ordered 1 to 72 with three rows and each row contained 24 plants. The plants were watered every five days during the dry season. After three weeks and every week thereafter for two months, the plants’ leaves were examined for fourth-instar nymphs. The fourth-instar *B*. *tabaci* nymphs were collected and preserved in 90% ethanol.

**Fig. 1:**
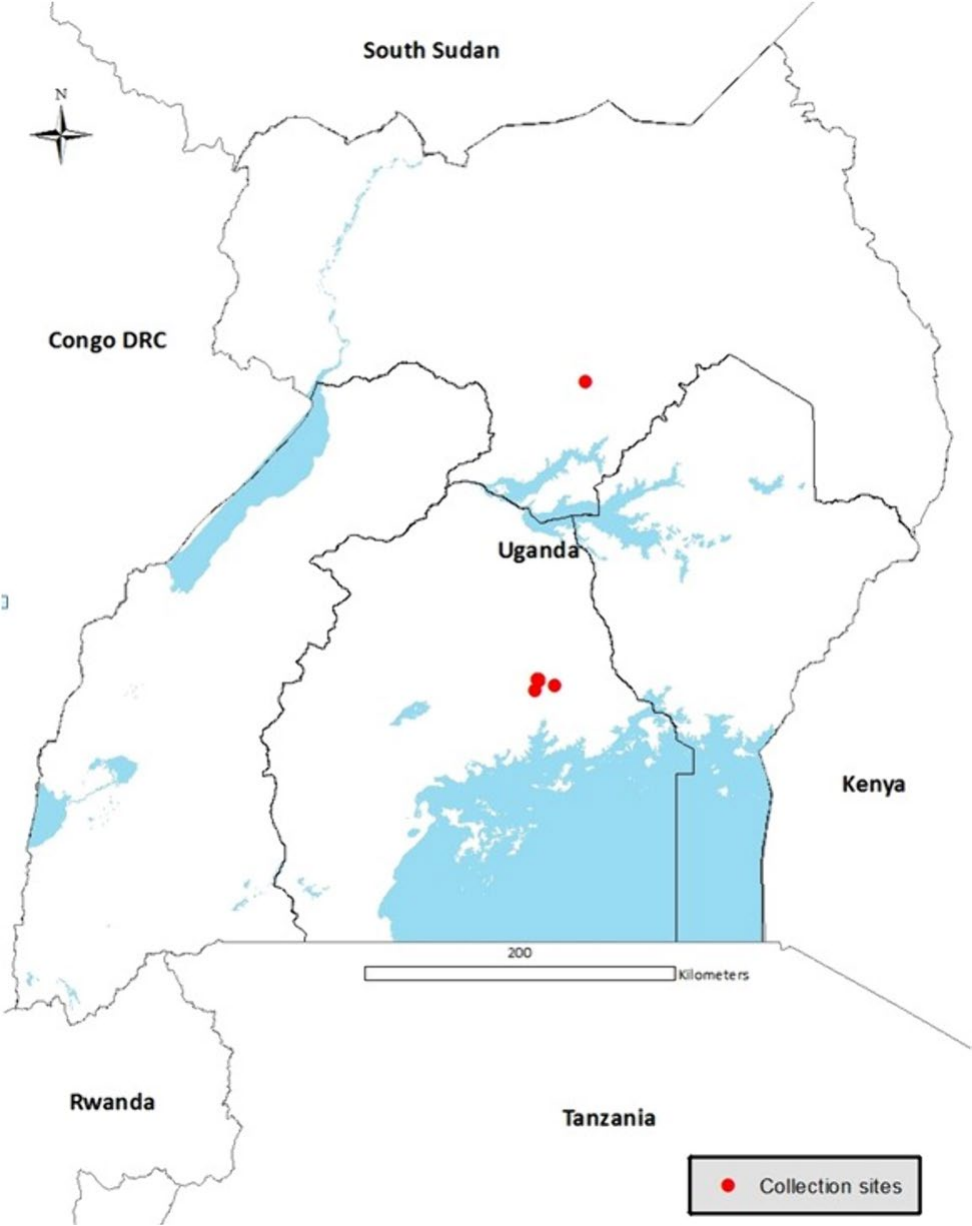
Locations (red circles) where fourth-instar nymphs were collected in 2017 from plants situated a distance of more than 300 m away from cassava

Whiteflies did not colonise *A*. *esculentus* (okra), *V*. *unguiculata* (cowpea) and* M*. *glaziovii* (tree cassava), in the experimental study area at Namulonge, but whitefly nymphs have been seen developing on these species elsewhere. We widened the search, therefore, to Lira for okra (N2.418294, E32.150063), Kabanyolo for cowpea (N0.466478, E32.610252) and Mukono for tree cassava (N0.435072, E32.768698) (Table 1), where these plant species could be found separated from cassava by distances greater than 300 m. Leaf samples for each host plant species were kept in separate plastic ‘Ziplock’ bags. Geo-coordinates (latitude and longitude) were recorded for each collection site using a geographical positioning system (GPS, Garmin eTrex Vista Cx) and used to generate a map (Fig. 1). All samples were collected during the second rainy season of 2017 (August to December).

### Growing plants

Plant species and whitefly colonies (Table 1) were raised in an insect-proof screen house at NaCRRI, Namulonge at 27 ± 5 °C, 70 ± 5% relative humidity (RH) and 12:12 light: dark photoperiod (L:D). Plants were raised on sterilised forest soil mixed with chicken manure at a ratio of 10:3. The soil mixture was sterilised by steaming overnight and allowed to cool before use. At three fully expanded leaf growth stage, plants were individually placed in Lock and Lock (LL) whitefly-proof cages (Wang et al. 2011). The LLs were modified with two additional side openings in the upper container covered by 160-µm nylon mesh for good ventilation. At the Natural Resources Institute, UK, two substrates: Multipurpose Jiffy substrates (Jiffy products, UK) and John Innes No. 2 compost (John Innes manufacturing association, UK), were sterilised by freezing for three days and mixed in a 1:1 ratio. The sterilised mixture was used to grow the different host plants that were propagated by seed (Table 1) and eggplant cv. Black Beauty. They were planted in potting cups (7 × 7 × 8 cm) and thereafter transferred to the growth room. The plants were raised and maintained in the growth room under a controlled environment of 25 ± 2 °C, 65 ± 5% RH, and 14:10 h L: D. Cassava, sweet potato, *M*. *glaziovii* and *C*. *benghalensis* cuttings were treated with a systemic insecticide and fungicide and planted in potting cups (7 × 7 × 8 cm) in the glasshouse. Plants were kept under quarantine in the glasshouse for two months before use in experiments. Experimental plants with three fully expanded leaves were also enclosed in LL cages.

### Rearing whiteflies

From each of the selected plant species, leaf samples with fourth-instar nymphs were collected from plants at least 300 m from cassava and kept in separate plastic ‘Ziplock’ bags. The samples were placed in petri dishes for adults to emerge. The emerged whiteflies were used to establish a live pure colony (one male and one female per plant), on the same host plant species from which they collected, individually enclosed in LL cages. The pure colonies were kept in LL cages for 2–4 months and then transferred to BugDorm whitefly-proof insect-rearing cages (60 cm × 60 cm × 60 cm) containing two or three plants.

The raised whiteflies were transferred to NRI, UK, for molecular analysis and host plant transfer experiments. Briefly, a day before the transfer, five leaves with fourth-instar nymphs were collected from each *B. tabaci* colony at NaCRRI, Namulonge and these were placed in petri dishes (90 × 15 mm). The petri dishes were sealed with parafilm, wrapped in cloth, placed in perforated polythene bags, and carefully packed in a box for shipping.

Upon arriving at NRI, emerged adults were released onto different egg plants (cv. Black Beauty) in LL cages and monitored in the controlled environment room at 26 ± 3 °C, 65 ± 5% RH and 12:12 h L:D. The whitefly colonies were kept in LL cages until their identity was determined. After 2–4 months, the colonies had built up to about 200 whiteflies per LL cage. The colonies were then transferred to BugDorm whitefly-proof insect-rearing cages containing two or three plants (with at least five fully expanded leaves) of the same plant species, from which each colony had been collected.

### Suitability of cassava for feeding and oviposition by *B. tabaci* species

The suitability of cassava for feeding and development was measured in terms of survival and oviposition by the *B. tabaci* species that were successfully established at NRI (Table 1). The SSA1-Hoslundia (Namuddu et al. 2023) was also included in the study to investigate whether it was similar to SSA1-SG1 and could develop on cassava. The SSA1-SG1 cassava colony that was collected from Kayingo in Uganda in 2016 and established at NRI, UK (Mugerwa et al. 2018), was used as the control. Cassava plants cv. (NAROCASS1, Magana and Columbian) with three fully expanded leaves were placed in LL cages. NAROCASS1 is an improved variety that is resistant to cassava mosaic disease (CMD) and tolerant to cassava brown streak disease (CBSD), but is susceptible to whiteflies. Magana is a Ugandan landrace that is susceptible to CMD and CBSD, but relatively resistant to whiteflies (Omongo et al. 2012), while Columbian variety is the standard used for rearing cassava whiteflies at NRI, UK. The experimental design involved four replicates and a random number was assigned to each plant. Twenty-five pairs of five-day-old, non-sexed adults were collected from colonies that had been maintained on their respective host plants for at least four generations. The adults were taken from the apical leaves with the aid of an aspirator and introduced onto cassava plants. Surviving adults were counted daily until the eighteenth day after release, when early fourth-instar larvae were observed for SSA1-SG1. All instar stages and any eggs (dead or alive) were counted on each plant, with the aid of a hand lens (× 40), to determine the total *B*. *tabaci* eggs oviposited during this period. After counting, plants were kept for a further month to observe any adult emergence of *B*. *tabaci* EA1, MEAM1, MED-ASL, SSA1-Hoslundia, SSA6 and SSA12 on cassava. The experiments were carried out under controlled conditions (26 ± 3 °C, 65 ± 5% RH and 12:12 h L:D).

### Extraction of DNA from an individual whitefly nymph

Genomic DNA was extracted from two individual fourth-instar nymphs randomly picked from each sample (Eppendorf tube), using a Chelex method (White et al. 2009). Each whitefly in 75 µL of 10% Chelex^®^ (Sigma-Aldrich, St Louis, USA) containing 6 µL of 10 mg/mL proteinase K was crushed in a 1.5-mL Eppendorf tube with zirconium oxide beads using a bullet blender homogeniser (Next Advance, Averill Park, NY). The Eppendorf tubes with the extract were centrifuged for 1 min at 15,871 g. The extract was then incubated for one hour at 37 °C and then for 10 min at 96 °C to denature the enzyme. The DNA extracts were stored at − 20 °C until use.

### Polymerase chain reaction (PCR) of mtCO1 DNA, purification and sequencing

The 3’ region of the *mtCO1* gene was amplified using a forward primer, 2195Bt (TGRTTTTTTGGTCATCCRGAAGT), and reverse primer C012/Bt-sh2 (TTTACTGCACTTTCTGCC) with an expected amplicon size of 867 bp (Mugerwa et al. 2018). The total reaction mixture of 30 µL contained 2 µL of the DNA extract, 0.2 µM of each primer pair, 6 µL of 5X MyTaq Buffer HS (Bioline), 1.0 U of MyTaq Polymerase (Bioline) and molecular grade water (Sigma-Aldrich, UK). PCR cycling was performed in a Veriti 96-well thermal cycler (Applied Biosystems, UK) programmed as follows: Initial denaturation at 95 °C/3 min, 10 cycles of 95 °C/30 s, annealing at 45 °C/30 s and 72 °C/1 min; 20 cycles of 95 °C/30 s, annealing at 50 °C/30 s and 72 °C/1 min and final cycle at 72 °C/10 min. Electrophoresis of PCR products was done on 1% (w/v) agarose gels in 0.5 X Tris/borate/EDTA buffer, stained with RedSafe™ (iNtRON Biotechnology, Korea). PCR products on the agarose gel were visualised under UV light at 302 nm on a trans-illuminator GBOX, CHEMI 16 (Syngene, UK), and those with the expected size were purified using the exonuclease 1 and antarctic phosphatase (Exo1-AP) treatment (Werle et al. 1994) with slight modifications. The PCR products (20 µL) were mixed with 1 U Exo1 and 1 U AP (New England Biolabs, UK) and incubated for 20 min at 37 °C. The enzymes were inactivated for 10 min at 80 °C. Purified PCR products were sent for Sanger sequencing in both directions at GATC Biotech (Germany).

### Phylogenetic analysis of partial mtCO1 sequences

The partial mtCO1 Sanger sequences generated in this study were first screened using quality checks in Geneious version 10.2.3. Firstly, a consensus sequence was generated using the reverse and forward sequences for each individual whitefly (Kearse et al. 2012). The new consensus sequences of this study were aligned with high-throughput sequencing (HTS)-derived full mitogenome sequences downloaded directly from GenBank, using MAFFT (Katoh and Standley 2013). All Sanger sequences which contained indels were eliminated and not considered for further analysis. The remaining Sanger sequences together with HTS-sequences were then trimmed to 657 bp and translated to amino acid residues from appropriate codon positions using the invertebrate mitochondrial DNA genetic codes to (i) identify potential premature stop codons and (ii) enable amino acid residue alignment against the HTS reference CO1 amino acid data set. Sanger sequences that had premature stop codons and amino acid substitutions in highly conserved regions as identified within the trimmed HTS reference CO1 gene set were eliminated. Then 29 unique haplotypes were extracted from the remaining sequences (*n* = 179) and aligned with equivalent reference whitefly sequences downloaded directly from the GenBank in accordance with Kunz et al. (2019). MEGA-X was used to determine the best substitution model. Phylogenetic analysis was done using BEAST2, which employs Markov Chain Monte Carlo (MCMC) sampling to approximate posterior probabilities of phylogenies (Green 1995). It also utilises the BEAGLE library, an application programming interface, and library of high-performance statistical phylogenetic inference (Ayres et al. 2012). BEAUti version 2.5.1 was used to generate the *xml* file for analysis in BEAST version 2.5.1. Tree prior was set to 'Yule' with strict clock model and uniform birth rate. The site model was specified as TN93, base frequencies were estimated, and Gamma plus invariant sites was the site heterogeneity model. The MCMC was run for 10 million generations and sampled every 1000th generation (the first 10% of trees were discarded as burn-in). TreeAnnotator version 2.5.1 was used to generate a final tree and was visualised by FigTree version 1.4.3 to determine the similarities between reference sequences and haplotype sequences. *Bemisia afer* (accession no. KF734668) was used as the outgroup for the *B. tabaci* complex.

## Results

### Host plants utilised by *B. tabaci* nymphs in Uganda

**Fig. 2:**
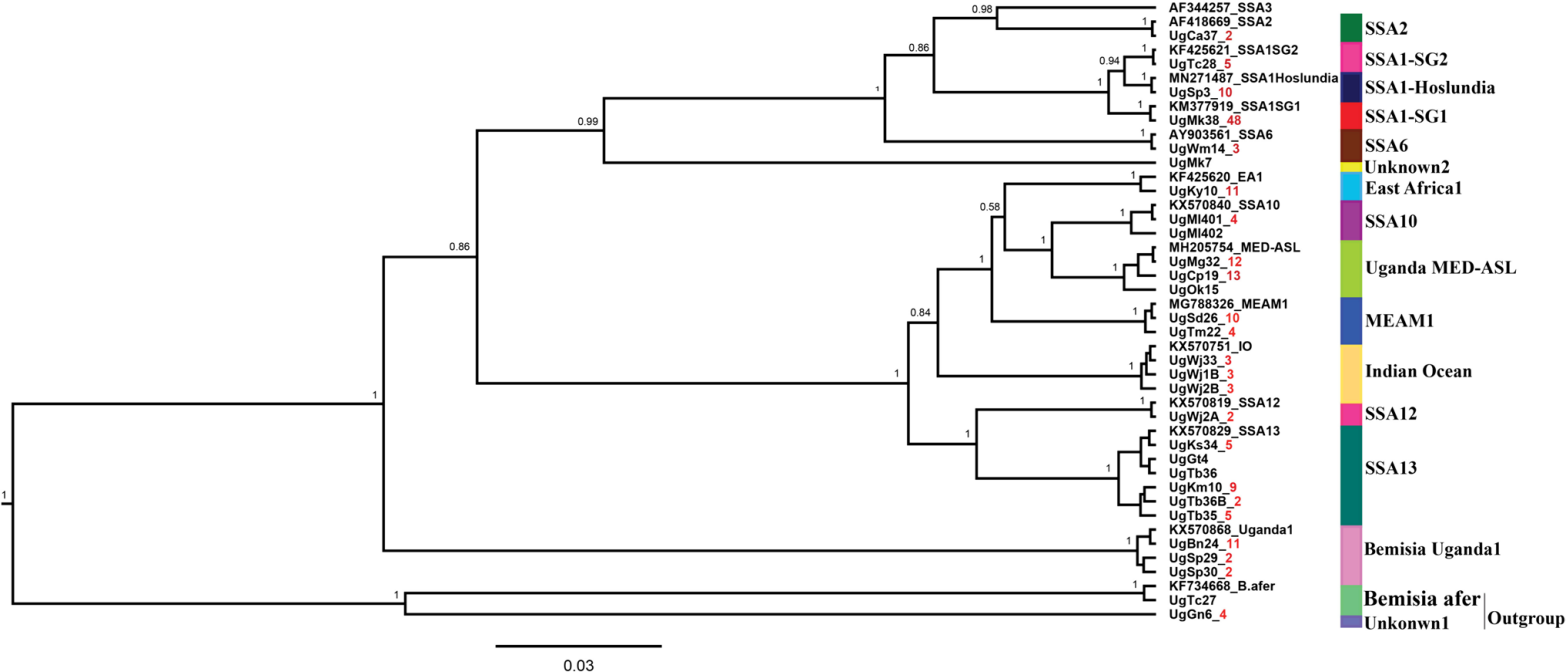
BEAST2 phylogenetic tree based on partial mtCOI sequences (657 bp) of individual fourth-instar whitefly nymphs collected from cassava and other host plant species within and adjacent to cassava, and plants at least 300 m away from cassava. Numbers noted at the nodes are posterior probabilities. Numbers in red besides a sample sequence are the number of individuals with that particular haplotype and with an asterisk (*) are reference sequences

Fourth-instar nymphs collected from cassava and common host plants in Uganda were identified based on phylogenetic analysis of partial mtCO1 sequences (657 bp) (Dinsdale et al. 2010) and are shown in Table 2. The whiteflies clustered with already published *B*. *tabaci* species and other whitefly species and they grouped into 14 genetically distinct groups (Fig. 2) that included: SSA1(SSA1-SG1, SSA1-SG2 and SSA1-Hoslundia), SSA2, SSA6, SSA10, SSA12, SSA13, EA1, IO, MEAM1, MED-ASL, *B*. Uganda1, *B. afer* and two new whitefly species. The results showed that *B. tabaci* species, SSA1 (SSA1-SG1 and SSA1-SG2) and SSA2 colonised cassava. However, the SSA1-Hoslundia, a subgroup of SSA1 did not colonise cassava, but utilised six other host plants, *H*. *opposita*,* A*. *africana*, *M*. *glaziovii*, *A*. *conyzoides*,* S*. *acuta* and *I*. *batatas* (Table 3). SSA1-SG1 utilised 11 alternative hosts whereas, SSA1-SG2 colonised alternative hosts, *E*. *heterophylla* and* M*. *glaziovii* (Table 3). SSA2 was not found on any of the alternative host plants sampled.

**Table 2:** The identities of fourth-instar *B. tabaci* and other whitefly species collected in the field and their percentage nucleotide similarity to closest relatives in the GenBank

*B*. *tabaci* and other whitefly species	Number of sequences	Closest GenBank accession no	References	Percentage nucleotide^§^
SSA1-SG1	48	KM377919	Ghosh et al. (2015)	100
SSA1-SG2	5	KF425621	Legg et al. (2014)	100
SSA1-Hoslundia	10	HE573751	Legg et al. (2014)	100
SSA2	2	AF418669	Maruthi et al. (2004)	100
SSA6	3	AY903561	Sseruwagi et al. (2005)	100
SSA10	5	KX570840	Mugerwa et al. (2018)	99.5–100
SSA12	2	KX570819	Mugerwa et al. (2018)	100
SSA13	23	KX570829	Mugerwa et al. (2018)	99.1–100
East Africa1	11	KF425620	Legg et al. (2014)	99.5
MEAM1	14	MG788326	Zein et al. (2018)	99.8–100
MED-ASL	26	MH205754	Vyskočilová et al. (2018)	99.2–100
Indian Ocean	9	KX570751	Mugerwa et al. (2018)	99.8–100
*Bemisia* Uganda1	15	KX570868	Mugerwa et al. (2018)	99.7–100
*Bemisia afer*	1	KF734668	Wang et al. (2014)	99.7
Unknown1	4	AF418673	Maruthi et al. (2004)	77.6
Unknown2	1	KF425619	Legg et al. (2014)	87.6

*SSA* Sub-Saharan Africa; *SG* subgroup; *MED* Mediterranean; *ASL* African silver leafing; *MEAM1* Middle East-Asia Minor1. ^§^ Percentage nucleotide identity with the closest GenBank sequence (%)

**Table 3:** Numbers of fourth-instar nymphs of *Bemisia tabaci* and other whitefly species collected from cultivated and non-cultivated plants in Uganda in 2017

Plant species	Plant family	SSA1			SSA2	SSA6	SSA10	SSA12	SSA13	EA1	IO	MEAM1	MED-ASL	B. Ug1	*B. afer*	Unknowns
		SG1	SG2	Hoslundia												
*Ageratum conyzoides*	Asteraceae	0	0	3	0	0	0	0	1	0	0	0	0	0	0	0
*Aspilia africana*	Asteraceae	5	0	1	0	0	0	0	0	0	0	0	1	2	0	1
*Bidens pilosa*	Asteraceae	2	0	0	0	0	0	0	0	2	0	0	0	2	0	0
*Vernonia amygdalina*	Asteraceae	5	0	0	0	0	5	0	0	0	0	0	0	0	0	0
*Commelina benghalensis*	Commelinaceae	0	0	0	0	0	0	2	3	0	7	0	0	0	0	0
*Ipomoea batatas*	Convolvulaceae	1	0	2	0	0	0	0	0	0	0	0	2	5	0	0
*Euphorbia heterophylla*	Euphorbiaceae	3	2	0	0	0	0	0	2	0	0	0	0	0	0	0
*Manihot esculenta*	Euphorbiaceae	11	1	0	2	0	0	0	0	0	0	0	0	0	0	0
*Manihot glaziovii*	Euphorbiaceae	6	2	1	0	0	0	0	0	0	0	0	0	0	1	0
*Arachis hypogaea*	Fabaceae	0	0	0	0	0	0	0	0	0	0	0	0	0	0	4
*Phaseolus vulgaris*	Fabaceae	0	0	0	0	0	0	0	0	2	0	3	0	5	0	0
*Vigna unguiculata*	Fabaceae	0	0	0	0	0	0	0	0	0	0	0	6	0	0	0
*Hoslundia opposita*	Lamiaceae	2	0	2	0	0	0	0	5	4	0	0	1	0	0	0
*Ocimum gratissimum*	Lamiaceae	1	0	0	0	3	0	0	0	0	0	0	0	0	0	0
*Abelmoschus esculentus*	Malvaceae	0	0	0	0	0	0	0	0	0	0	2	3	0	0	0
*Gossypium hirsutum*	Malvaceae	0	0	0	0	0	0	0	0	0	0	0	2	0	0	0
*Pavonia urens*	Malvaceae	1	0	0	0	0	0	0	0	1	1	0	8	1	0	0
*Sida acuta*	Malvaceae	0	0	1	0	0	0	0	3	0	1	1	3	0	0	0
*Phyllanthus niruri*	Phyllanthaceae	10	0	0	0	0	0	0	0	0	0	0	0	0	0	0
*Solanum lycopersicum*	Solanaceae	0	0	0	0	0	0	0	0	0	0	8	0	0	0	0
*Lantana camara*	Verbenaceae	1	0	0	0	0	0	0	9	2	0	0	0	0	0	0

Underlined binomials are cultivated host plants. *SSA* Sub-Saharan Africa; *SG* subgroup; *EA1* East Africa1; *IO* Indian Ocean; *MEAM1* Middle East-Asia Minor1; *MED* Mediterranean; *ASL* African silver leafing; *B. Ug1* *Bemisia* Uganda1; *B. afer* *Bemisia afer*

The whitefly species SSA6, SSA10, SSA12, SSA13, EA1, IO, MEAM1, MED-ASL, *B*. Uganda1, *B. afer* and two unknowns colonised non-cassava host plants. Most of them colonised more than two plant species except SSA6, SSA10, SSA12 and *B*. *afer*, which developed only on* O*. *gratissimum*, *V*. *amygdalina*,* C*. *benghalensis* and *M*. *glaziovii*, respectively (Table 3). One of the unknowns (Unknown1) that shared a maximum partial mtCO1 nucleotide identity of 77.6% to a known whitefly species, *B. afer* (accession no. AF418673) colonised *A*. *hypogaea* (Tables 2 and 3), while the second one (Unknown2) that shared only a maximum nucleotide identity of 87.6% to a published putative species, SSA2 (accession no. KF425619) utilised *A*. *africana* (Tables 2 and 3).

Host preferences were observed for the different cryptic species. SSA1-SG1 was closely associated Euphorbiaceae, Phyllanthacea and Asteraceae plant families, whereas MED-ASL mainly colonised the Malvaceae and Fabaceae families (Table 3). SSA1-SG2 was more common on Euphorbiaceae while SSA13 was more common on the Verbenaceae. MEAM1 was more prevalent on the Solanaceae. IO and SSA12 were more frequent on the Commelinaceae. Other cryptic species colonised particular plant species within a given plant family (Table 3).

### Distribution of whitefly species on different plants in the field

#### Whitefly species within cassava fields

**Fig. 3:**
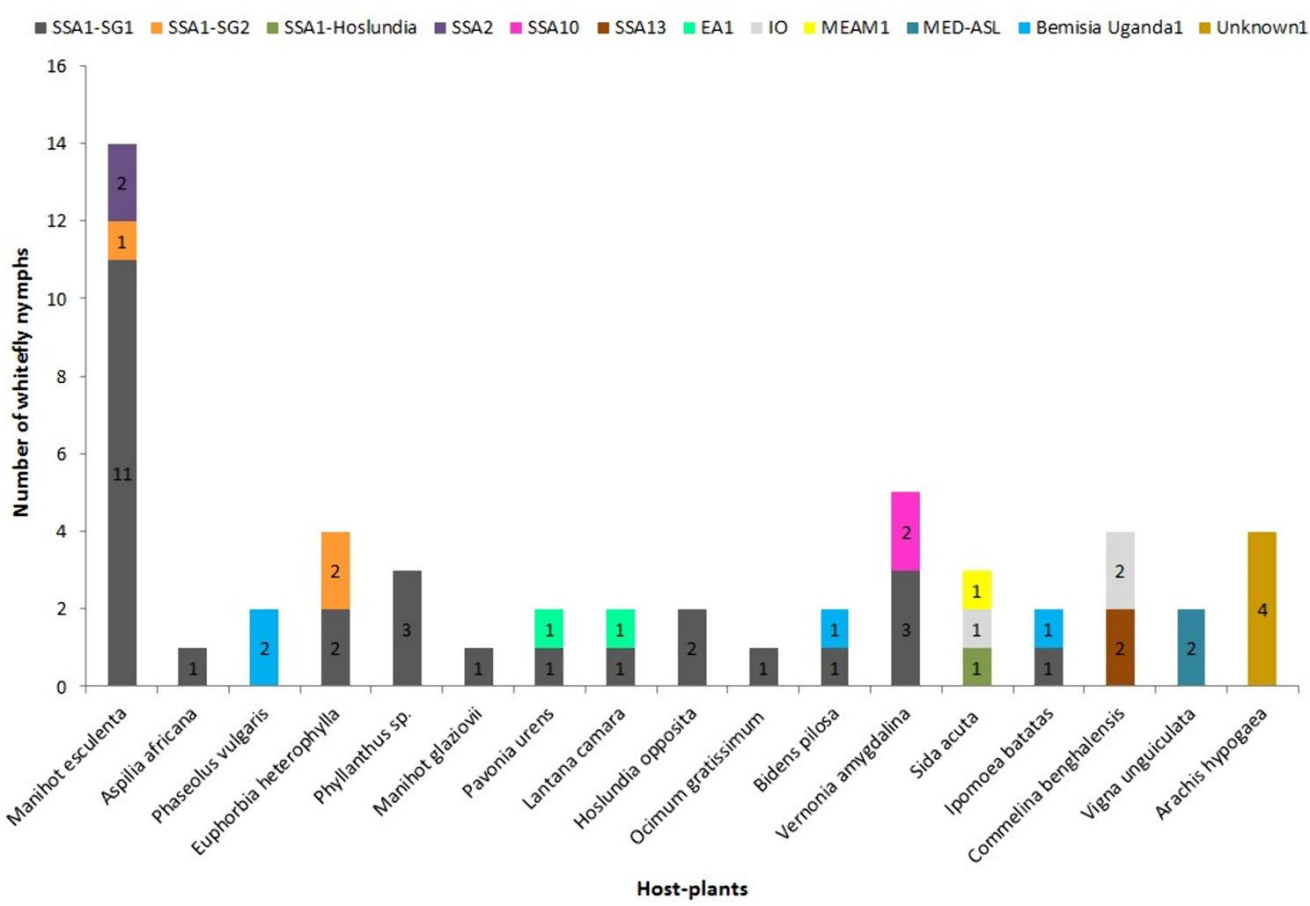
Distribution of whitefly species that colonised cultivated and non-cultivated host plants within cassava fields. *P*. *vulgaris*, *I*. *batatas*, *V*. *unguiculata* and *A*. *hypogea* were intercrops. SSA: sub-Saharan Africa, SG: subgroup, EA1: East Africa1, IO: Indian Ocean, MEAM1: Middle East-Asia Minor1 and MED-ASL: Mediterranean African silver leafing

Of the cassava *B*. *tabaci* identified in the study, SSA1-SG1 was the most predominant on cassava. It was more prevalent on cassava than on intercropped plant species and non-cultivated plants within cassava fields (Fig. 3). SSA1-SG1 and SSA1-SG2 equally colonised *E*. *heterophylla*. SSA1-SG1 colonised most of the host plants found within cassava fields except *P*. *vulgaris*, *S*. *acuta*, *C*. *benghalensis*, *V*. *unguiculata* and *A*. *hypogaea* (Fig. 3). MED-ASL and SSA12 colonised only *V*. *unguiculata* and *C*. *benghalensis*, respectively. At least two whitefly species colonised the same plant species within cassava fields, except MED-ASL and Unknown1, which utilised *V*. *unguiculata* and *A*. *hypogaea*, respectively (Fig. 3).

### Whitefly species that colonised host plants adjacent to cassava fields

**Fig. 4:**
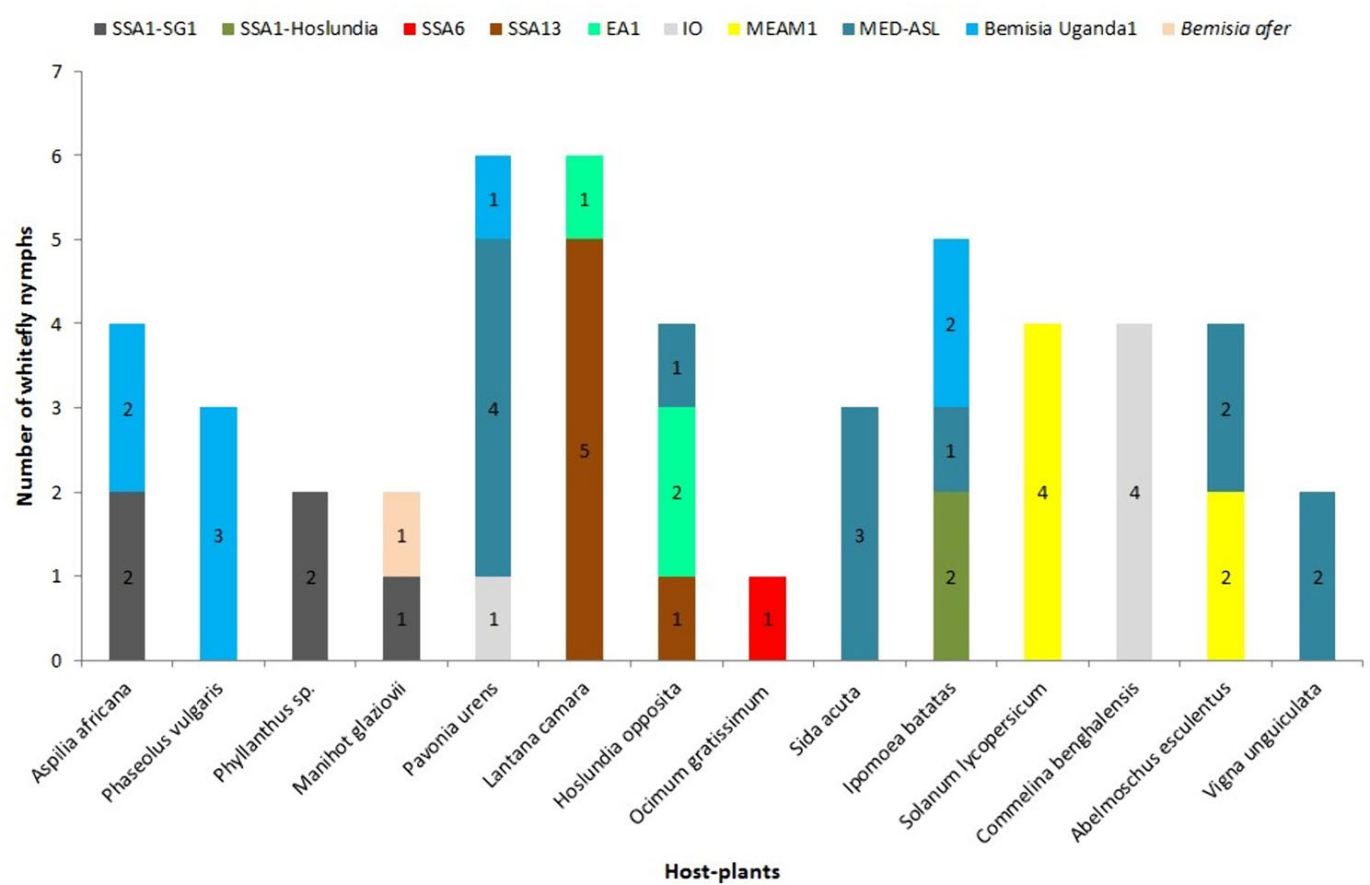
Distribution of whitefly species that colonised cultivated and non-cultivated host plants adjacent to cassava fields. *P*. *vulgaris*, *I*. *batatas*, *S*. *lycopersicum*, *A*. *esculentus* and *V*. *unguiculata* were cultivated plant species. SSA: sub-Saharan Africa, SG: subgroup, EA1: East Africa1, IO: Indian Ocean, MEAM1: Middle East-Asia Minor1 and MED-ASL: Mediterranean African silver leafing

SSA1-SG1 was the only cassava *B*. *tabaci* that colonised alternative host plants adjacent to cassava. It utilised three out of the 14 host plants sampled, all of which were non-cultivated (Fig. 4). MED-ASL was the most polyphagous species and it was found on six host plants, three of which were cultivated. *B*. Uganda1 colonised four host plants. SSA13, EA1, IO, MEAM1 each colonised two host plants and MEAM1 utilised only cultivated host plants (Fig. 4). SSA1-Hoslundia, SSA6 and *B*. *afer* colonised only one host plant (Fig. 4).

### Whitefly species that colonised host plants more than 300 m from cassava fields

**Fig. 5:**
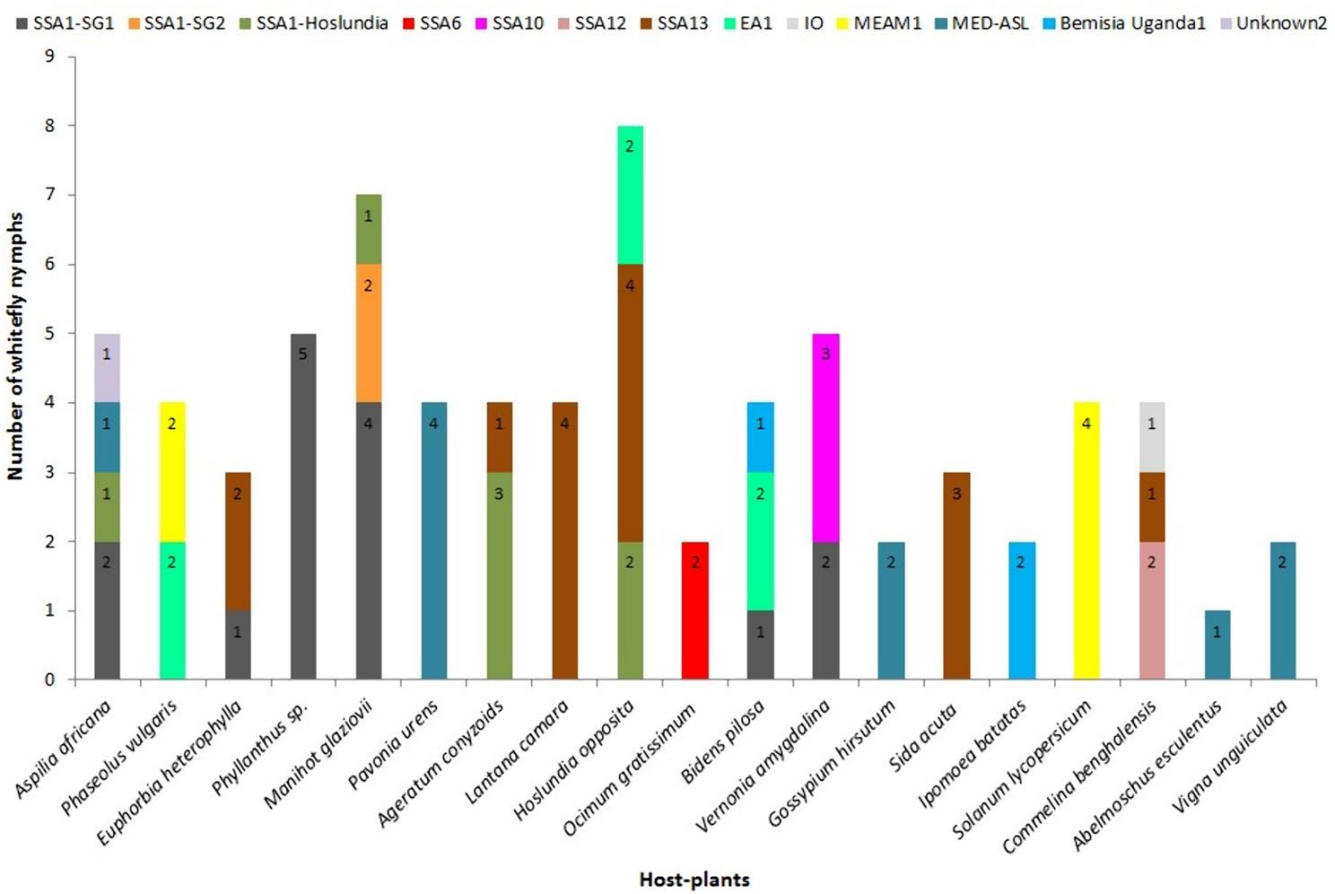
Distribution of whitefly species that colonised cultivated and non-cultivated host plants more than 300 m from cassava fields. Nymphs on *A. africana*, *P*. *niruri*, *H*. *opposita*, *O*. *gratissimum*, *V*. *amygdalina*, *L*. *camara*,* E*. *heterophylla* and *S*. *acuta* were collected from the bush; *A*. *esculentus*, *M*. *glaziovii* and *V*. *unguiculata were* collected from farmers’ fields in Lira, Mukono and Kabanyolo, respectively; and the rest were from the experimental plot at NaCRRI.* P*. *vulgaris*, *G*. *hirsutum*, *I*. *batatas*, *S*. *lycopersicum*, *A*. *esculentus* and *V*. *unguiculata* were cultivated plant species. SSA: sub-Saharan Africa, SG: subgroup, EA1: East Africa1, IO: Indian Ocean, MEAM1: Middle East-Asia Minor1 and MED-ASL: Mediterranean African silver leafing

SSA1-SG1, SSA1-Hoslundia, and SSA1-SG2 colonised six, four and one host plant species, respectively, more than 300 m from cassava. All host plants colonised by the three whitefly species were non-cultivated (Fig. 5). EA1, MED-ASL and *B*. Uganda1 utilised both cultivated and non-cultivated plants, whereas MEAM1 colonised only cultivated host plants (Fig. 5). SSA6, SSA10, SSA12, SSA13 and IO developed on non-cultivated plant species (Fig. 5).

### Suitability of cassava for feeding and oviposition by *B. tabaci*

#### Adult survival

**Fig. 6:**
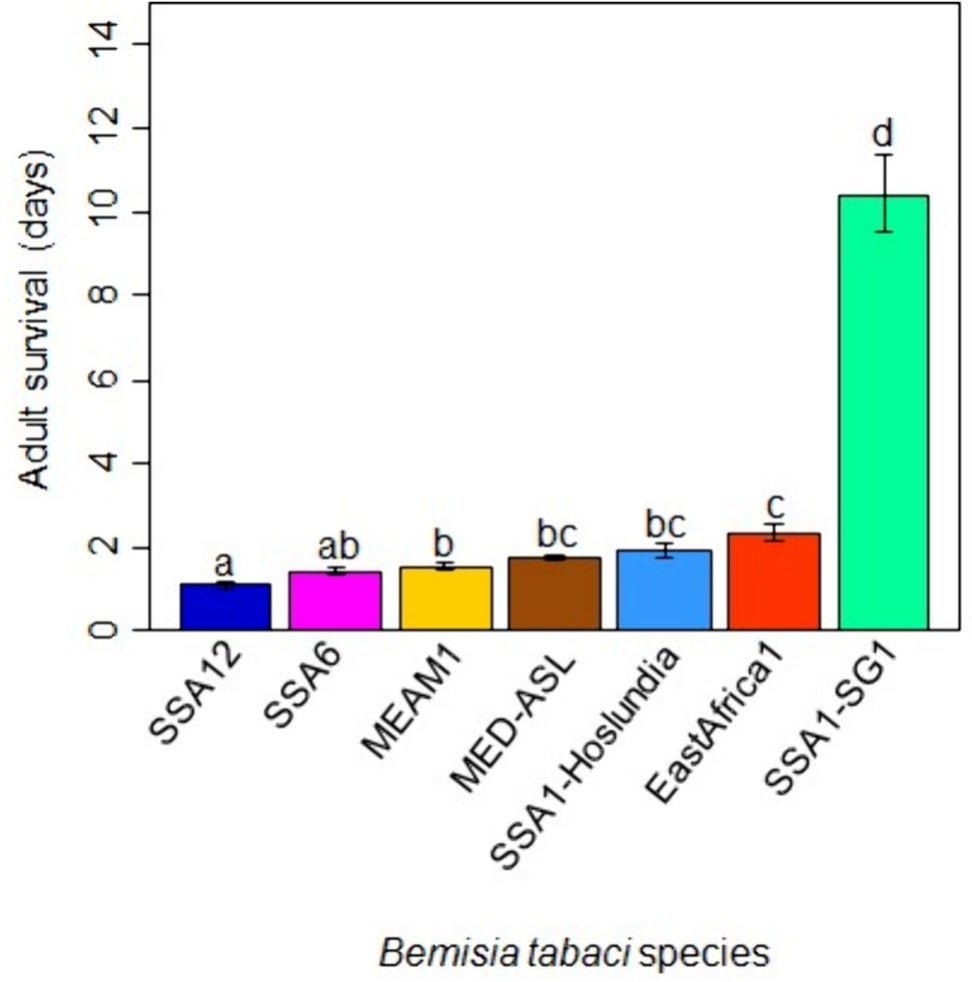
Survival of different *B. tabaci* species on cassava (data on the three cultivars pooled together), evaluated for 18 days. Error bars represent standard error of the means (*N* = 4). Different letters above the bars indicate significant differences (*P* < 0.0001). SSA: sub-Saharan Africa, MEAM1: Middle East-Asia Minor1, MED-ASL: Mediterranean African silver leafing and SG: subgroup

The suitability for adult feeding was measured in terms of average adult survival on cassava. Cassava cultivar had no significant effect on the adult survival of the seven *B. tabaci* species: EA1, MEAM1, MED-ASL, SSA1-SG1, SSA1-Hoslundia, SSA6 and SSA12 (*F* = 0.35, DF = 2, *P* = 0.70), and there was no significant interaction between cassava cultivar and *B. tabaci* species (*F* = 1.14, DF = 12, *P* = 0.34). The type of *B. tabaci* species affected adult survival significantly on cassava (*F* = 101.81, DF = 6, *P* < 0.0001; Fig. 6). SSA1-SG1 had the longest survival time (10.4 ± 1.8 d), while SSA12 had the shortest survival time (1.1 ± 0.3 d). Only SSA1-SG1 lived longer than 3 days on cassava.

#### Fecundity

**Fig. 7:**
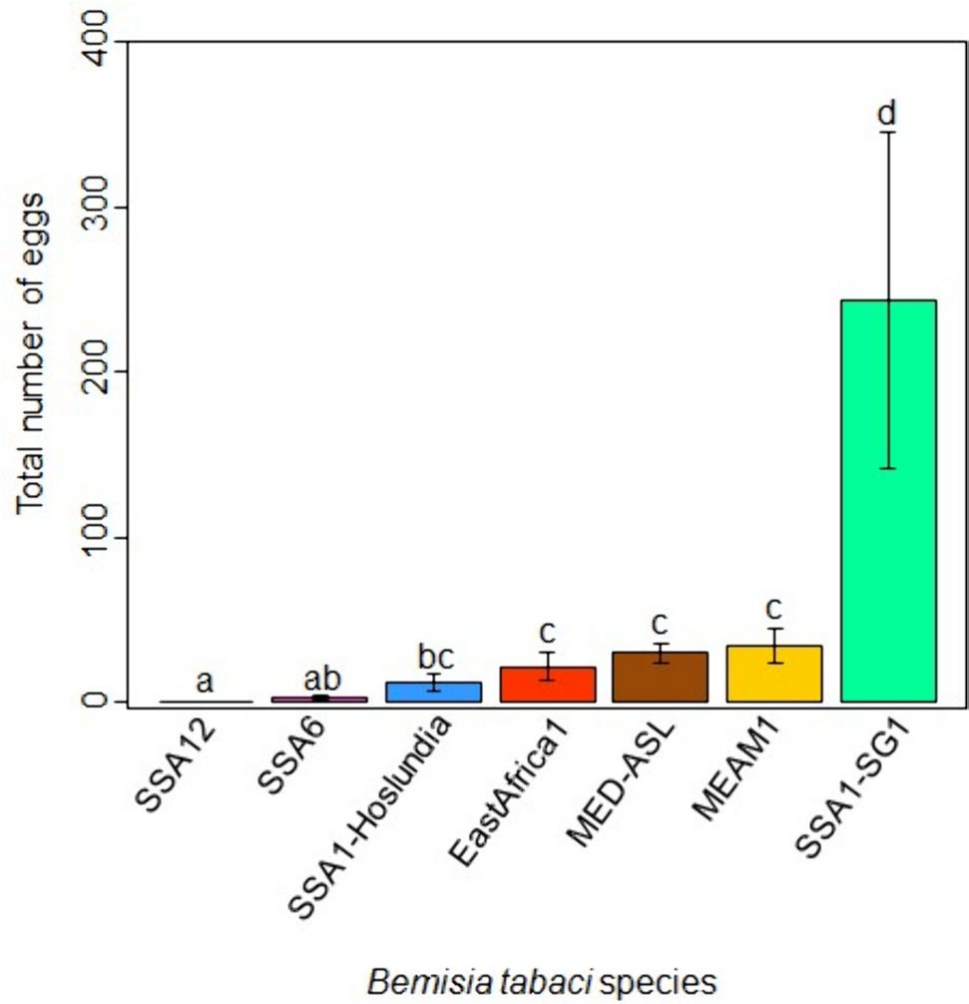
Number of eggs laid by different *B. tabaci* species in 18 days on cassava (data on the three cultivars pooled together). Error bars represent standard error of the means (*N* = 4). Different letters above the bars indicate significant differences (*P* < 0.0001). SSA: sub-Saharan Africa, MEAM1: Middle East-Asia Minor1, MED-ASL: Mediterranean African silver leafing and SG: subgroup

**Fig. 8:**
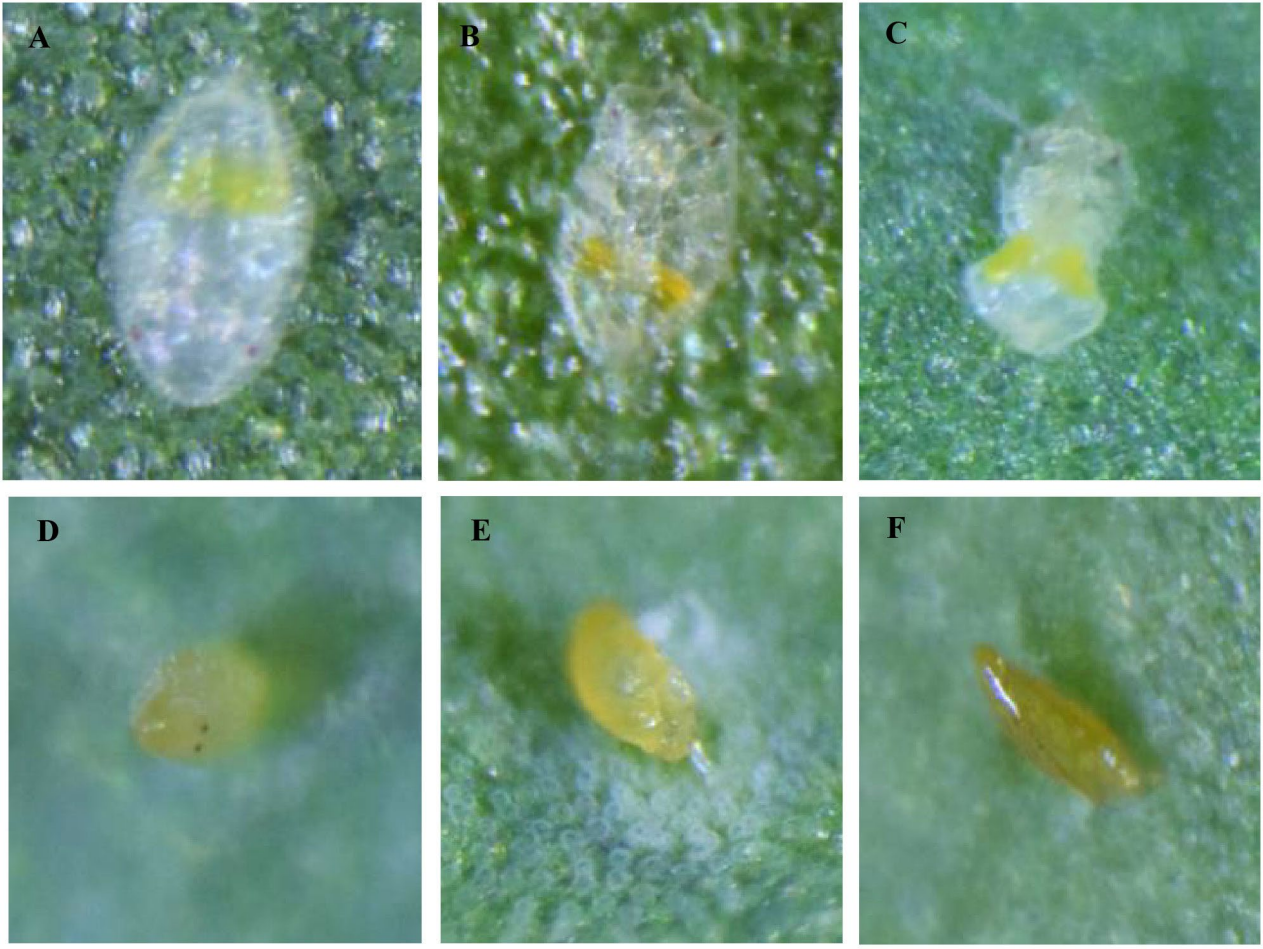
First-instar nymphs and eggs on cassava A: healthy first-instar, B: dehydrated first-instar, C: shrivelled first-instar, D: healthy egg, E: shrivelled egg, and F: dehydrated egg. The nymphs of non-cassava *B*. *tabaci* died at first-instar stage and the eggs that did not hatch were shrivelled or dehydrated. The photographs were taken at × 50 magnification on a stereomicroscope (Nikon SMZ18) with an attached DSLR camera (Nikon D5300) and illuminated with a LED light source (Photonic Optics F3000)

Cassava cultivar had no significant effect on the number of eggs laid by the different *B. tabaci* species (*P* = 0.55, GLM analysis of deviance) and no significant interaction occurred between cassava cultivar and *B. tabaci* species (*P* = 0.60, GLM analysis of deviance). *B. tabaci* species had a significant effect on the number of eggs laid on cassava (*P* < 0.0001, GLM analysis of deviance; Fig. 7). SSA12 laid the least number of eggs (0.3 ± 0.2), while SSA1-SG1 laid the highest number of eggs on cassava (243.7 ± 101.9). The nymphs for EA1, MEAM1, MED-ASL, SSA1-Hoslundia, SSA6 and SSA12 did not develop beyond the first-instar and eggs that did not hatch were shrivelled or dehydrated (Fig. 8).

## Discussion

This study comprehensively investigated host plants utilised by *B. tabaci* species especially for growth and development in order to understand the role that alternative host plants play in their population dynamics.

The findings indicate that in Uganda, cassava is only colonised by cassava *B. tabaci*, SSA1 (-SG1 and -SG2), and SSA2, which was consistent with previous studies on cassava-colonising *B. tabaci* (Legg et al. 2002, 2014; Sseruwagi et al. 2005, 2006). The very closely related SSA1-Hoslundia (a subgroup of SSA1 at mtCOI level), however, was not detected on cassava but colonised *A*. *africana*, *M*. *glaziovii*, *H*. *opposita*, *A*. *conyzoides*,* S*. *acuta* and *I*. *batatas*. *A*. *africana*, *H. opposita*, *A. conyzoides* and *S. acuta* are wild hosts and are considered to be medicinal, except *S. acuta*. The medicinal plants are often found on field edges that have been left by farmers to fallow or hedge barriers in cases where there no nearby shrubs or forests. Medicinal plants are of great importance to women who pick, process and sell them. *A*. *africana* is used to stop bleeding and to treat wounds (Komakech et al. 2019). *H*. *opposita* treats skin blisters, worms, diarrhoea and yellow fever (Namukobe et al. 2011). It is also used as a stomach cleanser and to treat postnatal pain and wounds. *A*. *conyzoides* is used to treat wounds and provides strength to pregnant women (Namukobe et al. 2011; Tugume et al. 2016). *M*. *glaziovii* was introduced into East Africa in the nineteenth century as a source of rubber (Munro 1983) but is currently used as an ornamental and hedge-barrier plant in Uganda. *I*. *batatas* is an important food crop in Uganda (Mwanga and Ssemakula 2011) that was introduced in East Africa in the sixteenth century (Grigg 1974). It is commonly intercropped with cassava by small scale farmers. It was hypothesised that the inability of some *B*. *tabaci* to colonise cassava could be due to the poisonous hydrogen cyanide (Legg 1996).

Hydrogen cyanide (HCN) is stored in an intact form as cyanogenic glucosides in the cell vacuoles of cassava but tissue damage such as insect feeding exposes cyanogenic glucosides to β-glucosidases, leading to the formation of HCN (Calatayud et al. 1994). However, SSA1-Hoslundia colonised* M*. *glaziovii*, a wild relative of cassava which also contains cyanogenic glucosides (Joseph et al. 2000), so this is unlikely to be the reason it did not colonise cassava. Studies have shown that wild relatives of cassava contain a higher cyanogenic content than cassava (Wang et al. 2014). SSA1-Hoslundia does not appear to have evolved the ability to colonise cassava. This may be due to the high sugar levels in the cassava phloem sap (Li et al. 2016; Yan et al. 2019), which may create a high osmotic potential that is unsuitable for SSA1-Hoslundia. Transcriptome studies revealed that a cultivated cassava variety (KU50) from South East-Asia had a significantly higher number of genes involved in sucrose transport than in a wild subspecies of cassava (W14) (Wang et al. 2014).

In addition to cassava, SSA1-SG1 colonised 11 plant species from seven families and these included: *A*. *africana*, *E*. *heterophylla*, *P*. *niruri*, *M*. *glaziovii*, *P*. *urens*,* L*. *camara*, *H*. *opposita*, *O*. *gratissimum*, *B*. *pilosa*, *V*. *amygdalina* and *I*. *batatas* while, SSA1-SG2 colonised *E*. *heterophylla* and* M*. *glaziovii*. These results complement findings by Sseruwagi et al. (2006) who reported that SSA1 colonised five alternative hosts, *M*. *glaziovii*, *J*. *gossypifolia*, *E*. *heterophylla*, *A*. *africana*, and *A*. *esculentus*. However, SSA1 species were not detected on *A*. *esculentus* in our study area. We also did not identify *J*. *gossypifolia* in the area where the study was carried out. MEAM1 and MED-ASL (recently called Uganda ‘ASL’) developed on *A*. *esculentus* in the field. MEAM1 has been reported to displace indigenous species in the Americas, Australia and China, particularly in areas where insecticides are used, due to its resistance to several groups of insecticides (Costa and Brown 1991; De Barro et al. 2006; Zang et al. 2006; Hu et al. 2011). Insecticide usage in our study area was limited and thus it could be that MEAM1 and MED-ASL are more specialised in utilising *A*. *esculentus* than SSA1. It has been hypothesised that insect herbivores may shift from one hostplant to another as result of competition (Gassmann et al. 2006). Therefore, SSA1-SG1 could have shifted from *A*. *esculentus* due to competition from MEAM1 and MED-ASL. SSA2 did not colonise the alternative hosts. This could be due to the replacement by SSA1-SG1 as earlier reported by Legg et al. (2014), leading to the low population that made it hard to be detected on other hosts.

The alternative host plants that were colonised by SSA1-SG1 were mainly wild hosts, of which* E*. *heterophylla* and* P*. *urens* are common weeds. *L*. *camara*, *A*. *africana*, *P*. *niruri*, *H*. *opposita*, *O*. *gratissimum*, *B*. *pilosa* and *V*. *amygdalina* are medicinal plants (Namukobe et al. 2011). Most of them are commonly found in the fallow borders, hedge-barriers, shrubs, or forests except *B*. *pilosa* and *Phyllanthus niruri*, which are common weeds in the gardens. *L. camara* treats ring worms and tuberculosis (Kirimuhuzya et al. 2009; Tugume et al. 2016). *B*. *pilosa* stops bleeding and treats wounds. *P*. *niruri* is used to treat measles, wounds, fever and stomach problems (Tugume et al. 2016).* O*. *gratissimum* is a remedy for stomach pain (Tugume et al. 2016).* V*. *amygdalina* cures malaria, stomach-ache and convulsions (Namukobe et al. 2011; Tugume et al. 2016). Although SSA1-SG1 was polyphagous, it did not colonise *A*. *conyzoides* and* S*. *acuta*, which were colonised by SSA1-Hoslundia. These clear differences in host plant utilisation between the two populations suggest that these could be separate species. This however, requires further investigation through reciprocal crosses.

SSA1-SG1 was more prevalent on cassava than alternative host plants commonly found in proximity to cassava, suggesting that it has a preference for cassava. Omondi et al. (2005) examined the host preference of cassava whiteflies in Ghana and revealed that they significantly chose cassava for oviposition and development than* A*. *esculentus*, *S*. *melongena*,* S*. *lycopersicum*, *S*. *aethiopicum* and *V*. *unguiculata*. In addition, SSA1-SG1 colonised 11 alternative host plants found in close proximity to cassava compared to > 300 m from cassava, where it colonised only six hosts. This implies that the high populations on cassava spread to nearby hosts including poor hosts such as *L*. *camara* (Namuddu et al., Unpublished data), whereas at a distance of > 300 m, SSA1-SG1 selects the most suitable hosts.

Cassava was introduced in East Africa in eighteenth century (Guthrie 1987). A study by Mugerwa et al. (2018) reported that Africa is the centre of origin of *B*. *tabaci*. In this study, 75% (9/12) of the host plants colonised by SSA1-SG1 were wild hosts, implying that these might be the native plants on which this species evolved. The strong preference for cassava may be the result of much more recent evolutionary change due to the intensive and continuous cultivation of cassava. Furthermore, six and one of the host plants colonised by SSA1-SG1 and SSA1-SG2, respectively, were found at least 300 m away from cassava, showing that these populations are capable of surviving on other plant species. These alternative hosts allow these whitefly species to survive in the absence of cassava and colonise, whenever it is planted. The high whitefly populations on cassava in Uganda could be due to the wide host plant range of SSA1-SG1. Studies have shown that the presence of alternative host plants provides a continuous breeding habitat for insect pests leading to increased populations (Joyce 1983; Sétamou et al. 2000; Johannesen and Riedle-Bauer 2014). Sétamou et al. (2000) reported that the abundant wild hosts for a snout moth, *Mussidia nigrivenella* Ragonot in Benin were responsible for its high numbers on maize during the cropping season. Similarly, Johannesen and Riedle-Bauer (2014) observed that a new host plant, *Urtica dioica* L. led to increased populations of a plant-hopper, *Hyalesthes obsoletus* Signoret on grapes in Austria.

The ability of SSA1-SG1 to utilise several alternative hosts increases the risk of transmitting viruses between these hosts and cassava. Findings by Amisse et al. (2019) showed that Cassava Brown Streak Virus (CBSV) infected *Zanha africana* (Radlk.) Exell and *Trichodesma zeylanicum* (Burm.f.) R.Br. that are commonly found near cassava fields in Mozambique. They also detected CBSV and Ugandan CBSV in *M*. *glaziovii*. In India, Rajinimala et al. (2009) identified the bitter gourd yellow mosaic geminivirus in cassava, *A*. *indica*, *C*. *sparsiflorus* and *M*. *coromandelianum*. The availability of various host plants for SSA1-SG1 may result in the transmission of unknown viruses to cassava that could be epidemiologically important. These host plants may also serve as reservoirs for cassava viruses.

This study found non-cassava *B*. *tabaci* species that colonised only non-cultivated plants and those that developed on both cultivated and non-cultivated plants. IO, SSA6, SSA10, SSA12 and SSA13 colonised only non-cultivated plant species. IO colonised *P*. *urens*, *S*. *acuta* and *C*. *benghalensis*. Contrary to our findings, IO (Ug7) was previously reported as a polyphagous species that colonised cultivated plant species namely, *P*. *vulgaris* and* G*. *hirsutum* and a weed species, *C*. *benghalensis* in Uganda (Sseruwagi et al. 2005). SSA6, SSA10 and SSA12 colonised only *O*. *gratissimum*, *V*. *amygdalina *and *C*. *benghalensis*, respectively. This study is in agreement with the findings of Sseruwagi et al. (2005) that SSA6 (Ug3) colonised only* O*. *gratissimum* in Uganda. EA1 colonised *H*. *opposita*, *P*. *urens*, *L*. *camara*, *B*. *pilosa* and a cultivated plant species, *P*. *vulgaris*. EA1 was first identified from a single adult in Tanzania (Legg et al. 2014), and here we report that it is polyphagous, colonising both cultivated and non-cultivated plants. However, its role as a vector requires further investigation. MEAM1 utilised *P*. *vulgaris*, *A*. *esculentus*, *S*. *lycopersicum* and a weed species, *S*. *acuta*, while MED-ASL colonised non-cultivated plants including *H. opposita, P. urens, S. acuta*, *A*. *africana* and cultivated plants such as *A*. *esculentus*, *V*. *unguiculata*, *I*. *batatas* and *G*. *hirsutum*. Our findings supplement previous studies in Uganda, which reported that MED-ASL (Ug4) was polyphagous and colonised *Cucurbita sativus*, *C*. *pepo*, *L*. *nepetifolia* and *P*. *urens*, whereas MEAM1 (Ug6) was oligophagous and developed on *A*. *esculentus* and *C*. *benghalensis* (Sseruwagi et al. 2005). Vyskočilová et al. (2019) also observed that MED-ASL (Uganda ‘‘ASL’’) developed well on *G*. *hirsutum*, *A*. *esculentus*, *I*. *batatas*, *S*. *melongena* and *C*. *pepo*.

The findings from our study revealed that the polyphagy of SSA1-SG1 and other *B*. *tabaci* like MED-ASL contributes to the high populations on cassava and other crops, respectively. Ability to utilise various host plants makes the survival of polyphagous *B*. *tabaci* more flexible and their control difficult.

García and Zerbini (2019) proposed that uncultivated wild hosts were the original hosts for majority of the causal viruses from which they were transferred to crops by polyphagous whitefly vector populations. The ability of some whitefly species (SSA1-SG1, MED-ASL and *B*. Uganda1) to colonise both crops and weeds could potentially increase the transmission of viruses between plants resulting in mixed infections. Recombinant begomoviruses have been detected in cassava (Zhou et al. 1997; Berrie et al. 2001) and the viruses often originate from other hosts, suggesting the ability of polyphagous vector populations to facilitate formation of novel viruses.

Our findings also showed that EA1, MEAM1, MED-ASL, SSA1-Hoslundia, SSA6 and SSA12 cannot develop on cassava. Adults of these cryptic species of *B*. *tabaci* did not survive on cassava longer than three days. The immatures did not develop beyond first-instar stage and the eggs that did not hatch were shrivelled or dehydrated. In a host transfer experiment, Legg (1996) observed that *B*. *tabaci* adults collected from *G*. *hirsutum* and *I*. *batatas* in the field (mtCO1 species identity was not determined) and established on these host plants, did not develop on cassava and none of the adults lived longer than two days. The first-instar nymphs also died soon after hatching. The shrivelled and dehydrated eggs were probably laid by dying females that could not make a slit in the leaf to insert the pedicel, which absorbs water and solutes from the plant for embryo development and eclosion (Buckner et al. 2002). Mortality of adults and first-instars was probably as result of failure to feed successfully due to the inability to penetrate the leaf cuticle or presence of toxic compounds in cassava phloem sap. Feeding studies conducted by Milenovic et al. (2019) using an electrical penetration graph (EPG) system showed that feeding by non-cassava *B*. *tabaci* MED on cassava was characterised by short total phloem ingestion periods (< 1 h) compared to that of the cassava *B*. *tabaci* SSA1-SG3, which was > 4 h. There was longer total xylem feeding for MED, suggesting that the whiteflies were not ingesting enough phloem sap and thus became dehydrated. Douglas (2006) reported that phloem-feeding insects encounter a high osmotic pressure created by sucrose in the phloem sap, which can lead to dehydration. The high sucrose levels in cassava (Wang et al. 2014) probably led to dehydration of the non-cassava *B*. *tabaci* adults and first-instar nymphs. In addition, cassava is a well-defended plant species that contains cyanogenic glucosides (McMahon et al. 1995) and flavonoids (Prawat et al. 1995) and possibly suitable for only adapted whitefly species.

Some adults of EA1, MEAM1, MED-ASL, SSA1-Hoslundia, SSA6 and SSA12 survived on cassava for at least one day, which increases the possibility of transmission of non-persistent plant viruses that require only insect probing (Polston et al. 2014). When searching for suitable hosts, phloem-feeders make shallow probes to detect the carbohydrate content and presence or absence of secondary metabolites (Walling 2008). Milenovic et al. (2019) observed that during a 12 h EPG recording period, MED whiteflies were able to ingest cassava phloem sap for 41 min.

## Conclusions

The data obtained in this study showed that cassava in Uganda is only colonised by cassava *B. tabaci i*.*e*. SSA1-SG1, SSA1-SG2, and SSA2. The findings also indicate that the host range of SSA1-SG1 is much wider than that of other cassava-colonising *B. tabaci* species in Uganda, which plays an important role in its predominance. Its ability to colonise other plant species facilitates its survival during periods when cassava is not available. It could also acquire other whitefly-transmitted viruses, which are not known to infest cassava and may be of epidemiological importance. The alternative hosts may also act as reservoirs for known cassava viruses.

This study provides important information for making decisions on pest management practices. For instance, when making choices of cultural practices, cassava should not be intercropped with *I*. *batatas* because it may facilitate the multiplication and spread of SSA1-SG1 in the field. Weed management is a measure that can be used to reduce the role of wild host plants on the population build-up of SSA1-SG1. However, farmers might be reluctant to remove medicinal plants that are of great importance to them. Therefore, whitefly-resistant varieties would be a more cost-effective and sustainable option. Non-cassava *B*. *tabaci*, MED-ASL was polyphagous in Uganda, colonising horticultural crops, food crops and weeds, which makes it a threat to agricultural productivity. EA1 was also polyphagous, utilising cultivated and non-cultivated plant species. However, its role as a vector requires further investigation.

### Additional information

The 29 unique haplotype sequences of whiteflies identified in this study were deposited in the GenBank sequence database (Accession numbers PP554199–PP554227).

## Supplementary Information

Below is the link to the electronic supplementary material.

Supplementary file1 (DOCX 16 kb)

## Data Availability

Sequence data that support the findings of this study have been deposited in the GenBank sequence database of the National Center for Biotechnology Information (USA), Accession numbers PP554199–PP554227.
