# Supplementary material for: Is polyphagy of a specific cryptic Bemisia tabaci species driving the high whitefly populations on cassava in eastern Africa?
Source: J Pest Sci (2004). 2024 Sep 2;98(2):853–68. doi: 10.1007/s10340-024-01832-8 (PMC12062101; doi:10.1007/s10340-024-01832-8)
Supplement: Supplementary file 1 — Supplementary file1 (DOCX 16 kb) [file 10340_2024_1832_MOESM1_ESM.docx]

| No. | Plant species | Geo-coordinates |
| --- | --- | --- |
| 1 | *Aspilia africana* | N0.480898, E32.608737 |
| 2 | *Phyllanthus niruri* | N0.52838, E32.62324 |
| 3 | *Hoslundia opposita* | N0.523883, E32.632724 |
| 4 | *Ocimum gratissimum* | N0.520973, E32.628397 |
| 5 | *Vernonia amygdalina* | N0.520963, E32.62979 |
| 6 | *Lantana camara* | N0.480898, E32.608737 |
| 7 | *Euphorbia heterophylla* | N0.518842, E32.627445 |
| 8 | *Sida acuta* | N0.52094, E32.628418 |
| 9 | *Bidens pilosa* | N0.521257, E32.631322 |
| 10 | *Ageratum conyzoides* | N0.521257, E32.631322 |
| 11 | *Commelina benghalensis* | N0.521257, E32.631322 |
| 12 | *Manihot glaziovii* | N0.435072, E32.768698 |
| 13 | *Pavonia urens* | N0.521257, E32.631322 |
| 14 | *Vigna unguiculata* | N0.466478, E32.610252 |
| 15 | *Phaseolus vulgaris* | N0.521257, E32.631322 |
| 16 | *Gossypium hirsutum* | N0.521257, E32.631322 |
| 17 | *Solanum lycopersicum* | N0.521257, E32.631322 |
| 18 | *Abelmoschus esculentus* | N2.418294, E32.150063 |
| 19 | *Ipomoea batatas* | N0.521257, E32.631322 |

Table 1 Geo-coordinates for sites of host-plants from which whiteflies were collected >300m from cassava fields

Table 2 Geo-coordinates for sites of host-plants from which whiteflies were collected within cassava

| **No** | **Host plant** | **GPS Coordinates** |
| --- | --- | --- |
| 1 | Cassava | N0.522728, E32.625532  N0.520587, E32.632585  N0.518950, E32.627672  N0.526310, E32.626945 |
| 2 | *Euphorbia heterophylla* | N0.522728, E32.625532  N0.518950, E32.627672  N0.526310, E32.626945 |
| 3 | *Pavonia urens* | N0.522728, E32.625532 |
| 4 | *Phyllanthus niruri* | N0.522728, E32.625532  N0.518950, E32.627672  N0.526287, E32.627360 |
| 5 | *Bidens pilosa* | N0.518950, E32.627672  N0.526287, E32.627360  N0.528570, E32.624172 |
| 6 | *Commelina benghalensis* | N0.522401, E32.634278  N0.525492, E32.632962  N0.520178, E32.628942 |
| 7 | *Phaseolus vulgaris* | N0.526287, E32.627360 |
| 8 | *Ipomoea batatas* | N0.526310, E32.626945 |
| 9 | *Sida acuta* | N0.525492, E32.632962  N0.521957, E32.634378 |
| 10 | *Manihot glaziovii* | N0.528998, E32.619922  N0.527053, E32.618007 |
| 11 | *Ocimum gratissimum* | N0.528770, E32.620172 |
| 12 | *Vernonia amygdalina* | N0.508860, E32.616775 |
| 13 | *Vigna unguiculata* | N0.455582, E32.606037 |
| 14 | *Arachis hypogaea* | N0.522172, E32.631962 |
| 15 | *Aspilia africana* | N0.522908, E32.633538 |
| 16 | *Lantana camara* | N0.522235, E32.637908 |
| 17 | *Hoslundia opposita* | N0.521986, E32.638312 |

Table 3 Geo-coordinates for sites of host-plants from which whiteflies were collected adjacent to cassava

| No | Plant species | Geo-coordinates |
| --- | --- | --- |
| 1 | *Ipomoea batatas* | N0.522308, E32.633597  N0.522908, E32.633538  N0.523362, E32.637585 |
| 2 | *Hoslundia opposita* | N0.522308, E32.633597  N0.520587, E32.632585  N0.521988, E32.638312 |
| 3 | *Vernonia amygdalina* | N0.520587, E32.632585 |
| 4 | *Lantana camara* | N0.522017, E32.633498  N0.517633, E32.634263  N0.526513, E32.629848 |
| 5 | *Phaseolus vulgaris* | N0.521107, E32.634803  N0.521683, E32.636508 |
| 6 | *Sida acuta* | N0.526287, E32.627360  N0.526670, E32.629443 |
| 7 | *Pavonia urens* | N0.520587, E32.632585  N0.526670, E32.629443 |
| 8 | *Phyllanthus* *niruri* | N0.526670, E32.629443 |
| 9 | *Aspilia africana* | N0.521380, E32.638608 |
| 10 | *Manihot glaziovii* | N0.510747, E32.617280 |
| 11 | *Solanum lycopersicum* | N0.520628, E32.628972 |
| 12 | *Commelina benghalensis* | N0.521178, E32.628342 |
| 13 | *Abelmoschus esculentus* | N0.474242, E32.606785 |
| 14 | *Ocimum gratissimum* | N0.517465, E32.623752 |
